# American Women and Birth Control

**Published:** 1920-11-06

**Authors:** 


### American Women and Birth Control.

It is interesting to observe, in view of the discussions that have been carried on in these columns, that the New York State Federation of Women's Clubs has decided at its annual convention at Utica in favour of birth control. Prominent among those who supported the resolution were Mrs. Harry Lilly, president of the New York City Federation, and Mrs. Elmer Blair, past president, who called upon all members to "see their duty and do it." The resolution, according to the Central News Agency, urged the speedy removal of all barriers "due to legal restrictions, traditions, prejudice or ignorance, which now prevent parents having access to such scientific knowledge on the subject as is possessed by the medical profession."

Mrs. Blair said that physicians, nurses and scientists endorsed the resolution, and she added: "Women, there is nothing of more importance in the world, for the good of the world than this question." Catholic delegates, as might have been expected, were among the foremost opponents of the proposal. Their point of view was crystallised by one of them in the following declaration: "We are not living for this life alone. We are peopling the world not for the short time we are here, but for eternal life. We must not dare to usurp the power of God." Miss Goldsmith, a prominent war-worker, took up a sensible attitude when she expressed the opinion that it was a question for the physicians to decide, and that their decision should be sanctioned by women. Women knew, she added, the abuses of such knowledge.

Benjamin Franklin and Abraham Lincoln were cited as examples of the sons of poor parents who might have said: "We cannot afford to have children and educate them." But, in spite of strenuous opposition, the resolution was carried by 149 votes to 97, and an attempt on the following day to upset the decision was defeated. The action of these American women is chiefly significant as a sign of the times. It is almost certain that birth control is going to become one of the most hotly discussed practical questions of the near future.

